# Post-chikungunya arthritis: a longitudinal study in a tertiary care hospital in Bangladesh

**DOI:** 10.1186/s41182-022-00412-9

**Published:** 2022-03-08

**Authors:** Sigma Hossain, Minhaj Rahim Choudhury, Md. Ariful Islam, Md. Masudul Hassan, Surayea Yeasmin, Farzana Hossain, Mohammad Mostafa Zaman

**Affiliations:** 1 Department of Rheumatology, Bangabandhu Sheikh Mujib Medical University (BSMMU), Shahbag Avenue, Dhaka, Bangladesh; 2 Freelance Researcher, Dhaka, Bangladesh; 3 WHO Bangladesh, Dhaka, Bangladesh

**Keywords:** Chikungunya infection, Post-chikungunya arthritis, Health assessment questionnaire (HAQ), Longitudinal study, Bangladesh

## Abstract

**Background and objective:**

To identify the clinical patterns and consequences of post-chikungunya arthritis was the study's objective.

**Methods:**

This longitudinal study was carried out among 143 Chikungunya virus (CHIKV) infected adult patients at the rheumatology department, Bangabandhu Sheikh Mujib Medical University (BSMMU), Dhaka, Bangladesh, during the outbreak of CHIKV infection in 2017.﻿ The disease was categorized into three phases: acute or febrile (lasting up to 10 days), subacute (11–90 days), and chronic (> 90 days). Patients who progressed towards the chronic phase were followed up to 1-year. Post-CHIKV de novo chronic inflammatory rheumatisms (CIRs) were characterized by persistent mono or oligoarthritis, undifferentiated polyarthritis, or meet the criteria rheumatoid arthritis (RA) or Spondyloarthritis (SpA). In addition, functional status was assessed by the validated Bangla version of the Health Assessment Questionnaire (HAQ).

**Results:**

Mean age was 43.3 ± 11.5 years, and 51.0% were male. Within 1-year follow-up, 60 (41.9%) patients were suffering from arthralgia/ arthritis. Of them 52 patients did not have any pre-existing arthralgia/arthritis. 35 (65.3%) had undifferentiated arthritis, 10 (19.2%) had SpA, and 7 (13.5%) had RA. Patients with pre-existing rheumatological disorders, 6(4.2%) had SpA, 1(0.7%) had RA and 1(0.7%) had osteoarthritis. Polyarthralgia (n = 33, 55.0%) and polyarthritis (n = 20, 33.3%) were the main presentations. Female gender (OR: 0.45; CI: 0.21–0.96), positive IgG (OR: 0.30; CI: 0.12–0.76), and moderate to severe functional disability (OR: 3.46; CI: 1.62–7.40) were independent predictors of developing chronic post-CHIKV rheumatism.

**Conclusions:**

At 1-year follow-up, more than one-third of the patients remained symptomatic. Female gender, positive IgG, and moderate to severe functional disability contributed to the development of chronicity.

## Significance & innovations

- This is the first longitudinal study conducted on Chikungunya (CHIK) arthritis patients in a tertiary care hospital during the significant outbreak of CHIK in Bangladesh, and all patients were evaluated and diagnosed by rheumatologists.
- While most patients recover from acute chikungunya virus infection within days or weeks, some patients develop chronic and incapacitating joint morbidities due to this virus infection.
- Risk factors for developing chronic post-CHIKV rheumatism﻿ includes female gender, positive IgG, and moderate to severe functional disability contributed to the development of chronicity.

## Introduction

Chikungunya (CHIK) is a mosquito borne ( *Aedes* species) febrile illness caused by the Chikungunya virus (CHIKV). It was first identified during an epidemic of febrile polyarthralgia in Tanzania in 1953 [1]. Since then, outbreaks have been attributed to many Southeast Asia and Indian countries. The disease became known worldwide after a major epidemic occurred in 2005 on Reunion Island, France, when more than 30% of the population of this island was affected by Chikungunya fever (CHIKF) [2].

In 2008, the Institute of Epidemiology, Disease Control and Research (IEDCR), Bangladesh, and the International Centre for Diarrhoeal Disease Research, Bangladesh (ICDDR, B) identified and investigated the first outbreak Chikungunya fever in Rajshahi and Chapianawabganj districts of Bangladesh [3]. Two small-scale outbreaks were documented in rural communities in 2011 [4] and 2012 [5]. Dhaka, one of the most densely populated cities globally with approximately 18 million inhabitants [6], has experienced the Chikungunya outbreak in 2017.

Chikungunya virus (CHIKV) infection has an incubation period of 2–7 days [7]. CHIKF is characterized by an acute phase followed by subacute and chronic phases. Acute CHIKF is often accompanied by high fever, headache, maculopapular rash, myalgias, and severe arthritis/arthralgias. Exanthema and polyarthralgia primarily affect the hands and feet, which cause significant functional disabilities [8–10].

The subacute phase lasts between 11 days and 3 months. After 3 months, the chronic form can persist for 6 years [11]. Systematic reviews and meta-analyses had shown that approximately 25% of Chikungunya cases would develop chronic inflammatory rheumatism, and 14% would develop chronic arthritis [12]. McCarthy and Morrison cited age above 45, female gender, and the existence of prior musculoskeletal diseases as clinical-epidemiological risk factors for the chronicity of musculoskeletal complications in CHIKF [13].A South Indian study also represented similar risk factors [14]. In a small percentage of patients, a condition that resembles rheumatoid arthritis (RA) may develop, possibly caused by the presence of the HLA-DRB1 gene (associated with the development of RA) in these patients. However, unlike classic RA, high levels of rheumatoid factor and anti-cyclic citrullinated peptides are not detected [15]. The most common ultrasonographic findings were synovitis, tenosynovitis [16].

Several cross-sectional studies and case series have been reported in Bangladesh [17, 18]. Hossain et al. found joint pain and fever were common among the patients. Arthralgia was polyarticular type in 56.3% of the patients. About 83% of the patients reported low to very low overall quality of life. Nearly 30% of the patients lost more than 10 days of productivity due to severe arthropathy [19]. Unfortunately, no longitudinal study was done regarding Chikungunya infection, focusing on the enduring effect of the disease among the Bangladeshi population. Therefore, the present study had been undertaken to identify the clinical patterns and consequences of post-chikungunya arthritis.

## Materials and methods

### Study design

This longitudinal study was carried out among 143 CHIKV infected patients (IgM and/or IgG positive) at the outpatient department of Rheumatology, BSMMU, Dhaka, Bangladesh, during the outbreak of CHIKV infection in 2017. The study followed the Strengthening the Reporting of Observational Studies in Epidemiology (STROBE) guideline [20].

### Subject enrollment

Patients who experienced typical clinical symptoms of CHIKV infection (febrile illness with arthralgia/arthritis) during the peak of the recent Dhaka outbreak (May–September 2017) were enrolled in this study. For confirmed case, we followed the recommendations of the World Health Organization (WHO) [21] where during an established outbreak, a patient fulfilling laboratory criteria (detection of positive RT-PCR or IgM antibody and/or demonstration of rising titre of IgG antibody for CHIKV but not dengue) irrespective of the clinical presentation were considered as a confirmed case. On the other hand, patients who claimed to have CHIKV infection without physicians' confirmation (clinical and or laboratory) were excluded from the study. As a result, we had only confirmed cases. The disease was categorized into three phases: acute or febrile (lasting up to 10 days), subacute (11–90 days), and chronic (> 90 days) [22].

Post-Chikungunya- Chronic Inflammatory Rheumatism (pCHIK-CIR) was characterized by persistent mono or oligoarthritis, undifferentiated polyarthritis, or meet criteria for Rheumatoid Arthritis or Spondyloarthritis [23].

Patient who fulfills the American College of Rheumatology (ACR)/European League Against Rheumatism (EULAR) Classification Criteria 2010 criteria [24] was considered having rheumatoid arthritis.

A patient who fulfills Spondyloarthritis (axial and peripheral): Ankylosing Spondylitis Assessment Study (ASAS) criteria 2011 [25] and/or Ankylosing spondylitis: Modified New York Criteria 1984 was considered having Spondyloarthritis [26].

The term "undifferentiated arthritis" (UA) is used here to describe patients with early inflammatory arthritis, typically between six weeks and a year in duration, whose disease cannot yet be diagnosed or differentiated from other defined disorders. However, a diagnosis can often be determined within three months and only infrequently requires a year to become evident. Many such patients will eventually be diagnosed with rheumatoid arthritis (RA) after further evolution of the symptoms and findings [27].

### Patient assessment

Meticulous history taking and thorough physical examination were done on every patient after enrollment in the study. For each, 68 joints were examined. In addition, clinical, routine laboratory (complete blood count, CRP, etc.) and musculoskeletal ultrasonographic features were evaluated in BSMMU by a group of rheumatologists trained in musculoskeletal ultrasonography using the grayscale with a linear 15 MHz multifrequency transducer in B-mode and with power doppler (PD) (750 Hz PRF, low wall filter and gain adjusted just below the appearance of artifacts) (Machine: High-End Radiology Ultrasound System, Mode: Logiq 5 Pro [GE Healthcare]). Ultrasonography scanning technique, grayscale, PD machine setting, and definitions of abnormalities were standardized according to EULAR recommendations for reporting ultrasound studies in rheumatic and musculoskeletal diseases among investigators before the study [28].

Synovitis, tenosynovitis, and bursitis were defined according to Naredo et al. Synovial, tenosynovial, and intrabursal blood flow at each joint was evaluated PDUS. PD imaging was performed by selecting a region of interest that included the bony margins, synovial site, and a variable view of surrounding tissues [29]. When thickened median nerves found in ultrasound were accompanied by hand paresthesia, it was suggestive of carpal tunnel syndrome (CTS) [16].

Functional status was assessed by the validated Bengali version of the Health Assessment Questionnaire (HAQ) [30]. The HAQ-DI is scored from 0.0 (no functional disability) to 3.0 (marked functional disability), where 0.0 to 1.0 is none to mild functional disability, 1.1 to 2.0 is a moderate functional disability, and 2.1 to 3.0 is severe functional disability [31].

**Fig. 1:**
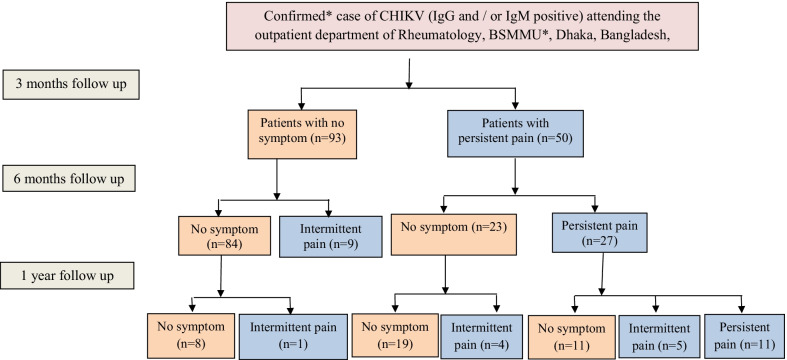
Study participants have been grouped into one image. *BSMMU Bangabandhu Sheikh Mujib Medical University**.** *The patient fulfilling the laboratory criteria {detection of positive reverse transcription-polymerase chain reaction or IgM antibody and/or demonstration of a rising titer of IgG antibody for Chikungunya virus (CHIKV) but not dengue}, regardless of the clinical presentation, is considered a confirmed case

Patients who have post-CHIK arthritis were followed up after one month and 3 months from the date of enrollment for documenting their clinical improvement. In addition, rheumatoid factor, ACPA, HLAB27, X-ray sacroiliac joint were performed according to the requirement of patient condition. Patients who progressed towards the chronic phase (more than three months) were followed up to one year (Fig. 1).

### Data analysis

Data were collected through a face-to-face interview in a pre-designed semi-structured questionnaire. Data were analyzed by using the SPSS-26 version. Descriptive statistical analysis included mean and standard deviation (SD) for continuous data and frequency and proportions for categorical data. For skewed data, a median with an interquartile range was used. While finding predictors of pCHIK-CIR, patients with intermittent or persistent pain after 3 months of acute pain were considered to have pCHIK-CIR. Associations of categorical data were assessed using the Chi-squared test, where *P* values less than 0.05 were considered significant. Crude odds ratios (OR) with 95% confidence interval (CI) were calculated. Gender, comorbidity, IgG, and functional disability were selected for the logistic model. Stepwise Logistic Regression was performed using backward elimination. Adjusted OR and 95% CI were calculated as part of the logistic procedure. It was mentionable that patients with pre-existing rheumatologic disorders were included in the logistic regression analysis as pre-existing rheumatologic diseases were found not to influence the pattern of joint involvement, type of arthritis, and pain characteristics on chronic patients.

### Ethical consideration

Study approval was obtained from the Institutional Review Board of BSMMU (BSMMU/2017/11262). Informed written consent was taken from the adult patients. A complete assurance was given that all information would be kept confidential. The right was being given to the patients not to participate and to discontinue participation at any time in the study with consideration/without penalty. The Declaration of Helsinki’s ethical guidelines were followed in the study.

### Results

The mean age of the patients was 43.3 ± 11.5 years, where 44 (30.8%) were from 31 to 40, and 43 (30.1%) were from the 41–50 years group. Seventy-three (51.0%) patients were male, 55 (38.5%) patients were service holders, and 53 (37.1%) were housewives. One hundred and thirty-six (95.1%) patients were urban residents. Comorbidity was present in 59 (41.3%) patients were 34 (23.8%) patients were hypertensive, and 30 (20.9%) were diabetic. The pre-existing rheumatologic disorder was present among 8 (5.6%) patients (Table1).

**Table 1:** Socio-demographic features of the patients (n = 143)

Socio-demographic features	*n* (%)
Age (in years)	
20–29	18 (12.6)
30–39	36 (25.2)
40–49	43 (30.1)
50–71	46 (32.2)
Gender	
Men	73 (51.0)
Women	70 (49.0)
Educational status	
No formal education (0)	15 (10.5)
Any primary education (1–5)	37 (25.9)
Any secondary education (6–10)	20 (14.0)
Higher secondary (11–12)	20 (14.0)
Above higher secondary (> 12)	51 (35.7)
Occupational status	
Service holder	55 (38.5)
Homemaker	53 (37.1)
Independent (businessman and day labourer)	26 (40.6)
Others (student and retired person)	9 (6.3)
Residential area	
Urban	136 (95.1)
Rural	7 (4.9)
Co-morbidity	59 (41.3)
Hypertension	34 (23.8)
Diabetes mellitus	30 (20.9)
Bronchial asthma	4 (2.8)
Hypothyroidism	4 (2.8)
Chronic kidney disease	1 (0.7)
Preexisting rheumatic disorders	
Pre-existing spondyloarthritis	6 (4.2)
Pre-existing rheumatoid arthritis	1 (0.7)
Pre-existing osteo-arthritis	1 (0.7)

Clinical features of the patients showed that all patients had a fever, 121 (84.6%) had symmetrical joint involvement, wherein 96 (67.1%) patients the involvement was generalized at the onset. Subsequently, ankle joint was mainly affected in 119 (83.2%) patients. Metacarpophalangeal (MCP) joint, wrist, Proximal interphalangeal (PIP) joints, and knee joints were affected in 100 (69.9%), 97 (67.8%), 84 (58.7%), and 76 (53.1%) patients, respectively. In 68 (47.6%) and 117 (81.8%) patients, joint swelling and morning stiffness were present, respectively. Myalgia was present in 47 (32.9%) patients, and skin involvement was present in 91 (63.6%) patients, where 67 (46.9%) patients had pruritic, erythematous rash. Forty-nine (34.3%) patients had moderate, while 15 (10.5%) patients had a severe functional disability. Laboratory findings showed that IgM was present in 135 (94.4%) patients, and IgG was present in 29 (20.3%) patients. Out of 143 patients, an investigation report for C-reactive protein (CRP) was present for 48 patients where 39 (81.2%) had high CRP (> 6 mg/l). Median ESR was 31.0 [15.0, 45.0] mm/hour (Table 2).

**Table 2:** Clinical features and laboratory findings of the patients in acute stage (*n* = 143)

Clinical features	*n* (%)
Fever	143 (100)
Symmetrical joint involvement	121 (84.6)
Joint involvement at onset	
Generalized	96 (67.1)
Ankle	22 (15.4)
Knee	15 (10.5)
Others	10 (6.9)
Subsequent joint involvement	
Ankle	119 (83.2)
Metacarpophalangeal joint	100 (69.9)
Wrist	97 (67.8)
Proximal interphalangeal joints	84 (58.7)
Knee	76 (53.1)
Shoulder	64 (44.8)
Elbow	51 (35.7)
Metatarsophalangeal joint	46 (32.2)
Distal interphalangeal joints	43 (30.1)
Others (hip, temporomandibular joint, axial plane)	30 (20.9)
Joint swelling	68 (47.6)
Morning stiffness	117 (81.8)
Myalgia	47 (32.9)
Skin involvement	91 (63.6)
Pattern of skin involvement	
Pruritic erythematous rash	67 (46.9)
Erythematous rash	24 (16.8)
Functional disability	
Mild	79 (55.2)
Moderate	49 (34.3)
Severe	15 (10.5)
Laboratory findings	
Positive Immunoglobulin M (IgM)	135 (94.4)
Positive Immunoglobulin G (IgG)	29 (20.3)
C-reactive protein (CRP) positive (*n* = 48)	39 (81.2)
Erythrocyte sedimentation rate (mm in 1st hour), median [IQR]	31.0 [15.0–45.0]
White blood cells count (10^3^/µL), mean ± SD	8.4 ± 2.4
Platelet count (10^3^/µL) mean ± SD	297.1 ± 91.7
Serum creatinine (mg/dl)	0.8 ± 0.2

*IQR Inter-quartile range

The total disability index of the patients was 1.4 [0.7, 1.9] where dressing had the lowest scores 1.0 [0.0, 1.0]. Walking, hygiene, and common daily activities had highest scores 2.0 [1.0, 2.0], 2.0 [1.0, 3.0] and 2.0 [1.0, 3.0] respectively (Table 3).

**Table 3:** The Health Assessment Questionnaire Disability Index (HAQ-DI) Scores of the patients in acute stage (*n* = 143), median [Q1,Q3]

HAQ-DI Scores	Median [Q1,Q3]
Dressing	1.0 [0.0, 1.0]
Arising	1.0 [0.0, 2.0]
Eating	1.0 [0.0, 2.0]
Walking	2.0 [1.0, 2.0]
Hygiene	2.0 [1.0, 3.0]
Reach	1.0 [1.0, 2.0]
Grip	1.0 [0.0, 2.0]
Common daily activities	2.0 [1.0, 3.0]
Total score	1.4 [0.7, 1.9]

**Fig. 2:**
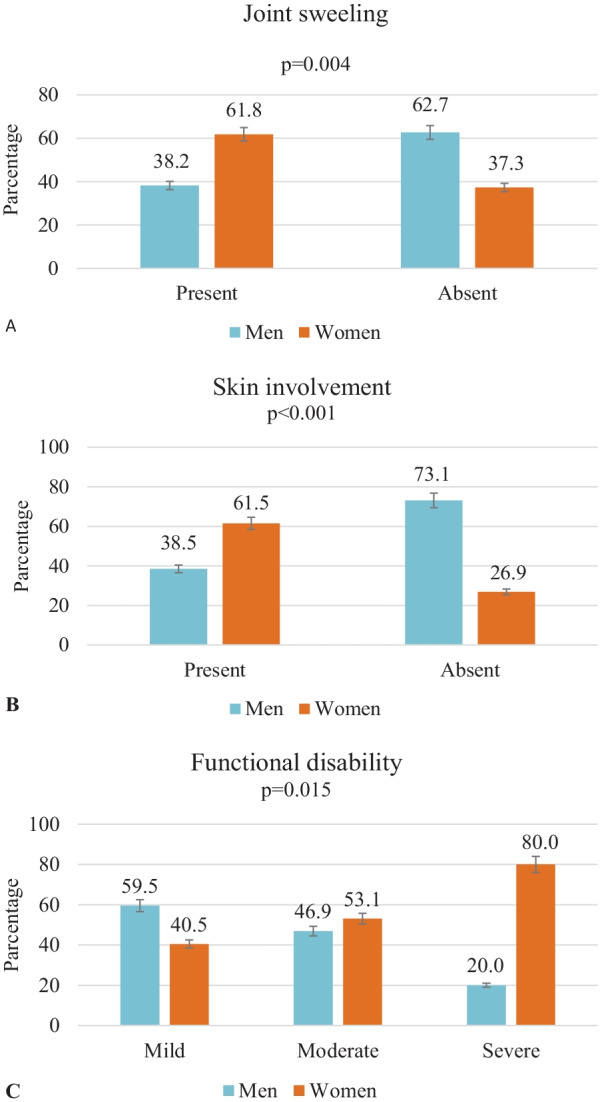
Joint swelling (**A**), skin involvement (**B**) and functional disability (**C**) of patients according to gender (*n* = 143)

Joint swelling (*p* = 0.004), skin involvement (*p* < 0.001), and functional disability (*p* = 0.015) were significantly more common in females (Fig. 2).

60 (41.9%) patients developed post-Chikungunya- Chronic inflammatory rheumatism (pCHIK-CIR) within the one-year follow-up. Pain without swelling was present in 39 (65.0%) patients. Ultra-sonographic findings showed that tenosynovitis was present in 19 (31.7%) patients, while median nerve entrapment was present in 4 (6.7%) patients. At the end of one year, HAQ score was available for 21 patients where 18 (85.7%) patients had a mild functional disability. No significant statistical difference was observed between patients with or without the pre-existing rheumatologic disorder (except tenosynovitis) and between males and females regarding characteristics of chronic patients (Table 4).

**Table 4:** Characteristics of the patients with Chronic Chikungunya arthritis (*n* = 60)

Characteristics	All (*n* = 60)	Preexisting rheumatic disorders		Gender	
		Absent (*n* = 52)	Present (*n* = 8)	Male (*n* = 26)	Female (*n* = 34)
Age (in years)					
Up to 40	27 (45.0)	23 (44.2)	4 (50.0)	14 (53.8)	13 (38.2)
> 40	33 (55.0)	29 (55.8)	4 (50.0)	12 (46.2)	21 (61.8)
Women	34 (56.7)	27 (51.9)	7 (87.5)		
Comorbidity	30 (50.0)	24 (46.1)	6 (75.0)	9 (34.6)	21 (61.8)
No preexisting rheumatic disorders	52 (86.7)			25 (96.1)	27 (79.4)
IgG positive	19 (31.7)	16 (30.8)	3 (37.5)	6 (23.1)	13 (38.2)
Type of joint involvement					
Only pain	39 (65.0)	34 (65.4)	5 (62.5)	18 (69.2)	21 (61.8)
Pain with swelling	21 (35.0)	18 (34.6)	3 (37.5)	8 (30.8)	13 (38.2)
Laboratory finding					
Anti CCP	1(1.7)	0 (0.0)	1 (1.9)	1 (3.8)	0 (0.0)
HLA B27	2 (3.4)	1 (1.9)	1 (1.9)	1 (3.8)	1 (2.9)
Anti CCP + HLA B27	1 (1.7)	1 (1.9)	0 (0.0)	1 (3.8)	0 (0.0)
RF	2 (3.3)	1 (1.9)	1 (1.9)	1 (3.8)	1 (2.9)
Ultra-sonographic findings					
Tenosynovitis	19 (31.7)	19 (36.5)	0 (0.0)	9 (34.6)	10 (29.4)
Synovial hypertrophy	8 (13.3)	8 (15.4)	0 (0.0)	2 (7.7)	6 (17.6)
Tendinitis	7 (11.7)	6 (11.5)	1 (1.9)	5 (19.2)	2 (5.9)
Median nerve entrapment	4 (6.7)	4 (7.7)	0 (0.0)	1 (3.8)	3 (8.8)
Joint effusion	3 (5.0)	2 (3.8)	1 (1.9)	1 (3.8)	2 (5.9)
Bursitis	2 (3.3)	2 (3.8)	0 (0.0)	1 (3.8)	1 (2.9)
X-ray finding					
Unilateral sacroiliitis	1 (1.7)	0 (0.0)	1 (1.9)	0 (0.0)	1 (2.9)
Bilateral sacroiliitis	4 (6.7)	4 (7.7)	0 (0.0)	1 (3.8)	3 (8.8)
Functional disability at one year	(*n* = 21)	(*n* = 17)	(*n* = 4)	(*n* = 9)	(*n* = 12)
Mild	18 (85.7)	15 (28.8)	3 (37.5)	8 (30.8)	10 (29.4)
Moderate	2 (3.3)	2 (3.8)	0 (0.0)	1 (3.8)	1 (2.9)
Severe	1 (1.7)	0 (0.0)	1 (1.9)	0 (0.0)	1 (2.9)

Numbers in parentheses indicated percentages. *CCP* Cyclic citrullinated peptide, *HLA-B27* Human leukocyte antigen B27, *RF* Rheumatoid Factor

Among the 60 CHIK patients, 35 had undifferentiated arthritis, where polyarthralgia was present in 21 (60.0%) patients, and polyarthritis was present in 8 (22.8%) patients. Ten patients had spondyloarthritis where polyarthralgia was present in 6 (60.0%) patients, and seven patients had rheumatoid arthritis where 5 (71.4%) had polyarthritis (Table 5).

**Table 5:** Types of arthritis and pain characteristics of the patients with Chronic Chikungunya arthritis (*n* = 60)

Type of arthritis	Oligoarthralgia *n* (%)	Polyarthralgia *n* (%)	Mono/Oligoarthritis *n* (%)	Polyarthritis *n* (%)
Undifferentiated Arthritis (*n* = 35)	2 (5.7)	21 (60.0)	4 (11.4)	8 (22.8)
Spondyloarthritis (*n* = 10)	0 (0.0)	6 (60.0)	1 (10.0)	3 (30.0)
Rheumatoid Arthritis (*n* = 7)	0 (0.0)	2 (28.6)	0 (0.0)	5 (71.4)
Preexisting Arthritis* (*n* = 8)	0 (0.0)	4 (50.0)	0 (0.0)	4 (50.0)

*Preexisting Arthritis** = **Pre-existing spondyloarthritis, Pre-existing rheumatoid arthritis, Pre-existing osteo-arthritis arthritis

**Fig. 3:**
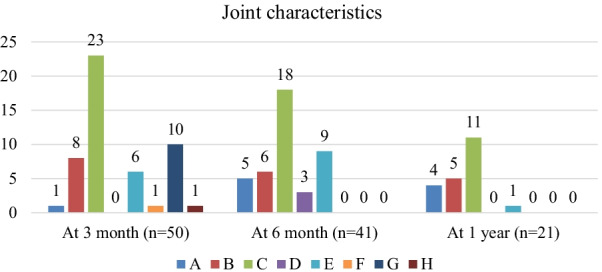
Trend of arthralgia and arthritis among patients with Chronic CHIK arthritis for one year follow up. *A* Monoarthralgia, *B* Oligoarthralgia, *C* Polyarthralgia, *D* Oligoarthritis, *E* Polyarthritis, *F* Oligoarthralgia & monoarthritis, *G* Polyarthralgia & oligoarthritis, *H* Polyarthralgia & Polyarthritis

At 3 months, in 23 (46.0%) patients, polyarthralgia and oligoarthritis were found in 10 (20.0%) patients. At 6 months, polyarthralgia was noted in 18 (50.0%) patients, and polyarthritis was reported in 9 (11.1%) patients. At one year, polyarthralgia was found in 11 (52.4%) patients, and only one patient (4.8%) had polyarthritis (Fig. 3).

Patients with comorbidity had significantly more chances of having pCHIK-CIR (Crude OR: 2.10; CI: 1.06–4.15, *p* = 0.039). Patients having positive IgG (Crude OR: 3.38; CI: 1.44–7.96, = 0.006) and moderate to severe functional disability (Crude OR: 0.34; CI: 0.17–0.69) also had significantly more chance of having pCHIK-CIR. Stepwise logistic regression using backward elimination showed that female gender (adjusted OR: 0.45; CI: 0.21–0.96, *p* = 0.039), positive Ig G (adjusted OR: 0.30; CI: 0.12–0.76, *p* = 0.011) and moderate to severe functional disability (adjusted OR: 3.45; CI: 1.62–7.40, *p* = 0.001)were independent predictor of developing pCHIK-CIR (Table 6).

**Table 6:** Predictors of post-Chikungunya- chronic inflammatory rheumatism (pCHIK-CIR)

Variables	Acute	Chronic	Crude OR	Adjusted OR
	*n* (%)	*n* (%)	(95% CI)	(95% CI)
Gender				
Men	47 (64.4)	26 (35.6)	Ref.	
Women	36 (51.4)	34 (48.6)	1.71 (0.87–3.34)	0.45 (0.21–0.96)*
Comorbidity				
Absent	55 (65.5)	29 (34.5)	Ref.	
Present	28 (47.5)	31 (52.5)	2.10 (1.06–4.15)*	0.62 (0.29–1.31)
IgG				
Negative	73 (64.0)	41 (36.0)	Ref.	
Positive	10 (34.5)	19 (65.5)	3.38 (1.44–7.96)*	0.30 (0.12–0.76)*
Functional disability				
Mild	37 (46.8)	42 (53.2)	Ref.	
Moderate to severe	46 (71.9)	18 (28.1)	0.34 (0.17–0.69)*	3.45 (1.62–7.40)*

*p < 0.05, *Ref.* Reference category, *OR* odds ratio. *CI* confidence interval

## Discussion

Our study is the first prospective cohort study among Bangladeshi CHIKV patients to identify post-Chikungunya arthritis's clinical patterns and consequences. We evaluated patients in all three phases (acute, sub-acute, and chronic) of the disease, for which we were able to identify the predictors of chronicity. The study population consisted of 143 CHIKV-infected patients, where 41.9% of patients developed pCHIK-CIR within the one-year follow-up, and polyarthralgia and mild functional disability were their main clinical presentation. In addition, female gender, positive IgG, and moderate to severe functional disability contributed to the development of chronicity.

More than half of the patients in the present study were male. Though female predominance was reported in several studies conducted in South India, Colombia, Venezuela, Sri Lanka [14, 32–36], male predominance was also reported in Singapore, Mayotte [37]. This inconsistency may relate to gender differences in exposure to infection due to community-specific habits, customs, or behaviors [38].

Most of the patients had a mild functional disability, while one-third had moderate and 10% had severe disability. This disability was significantly more in female patients. Female patients also had substantially more joint swelling and skin involvement than male patients, leading to more disability. This finding was comparable to the study of Kularatna et al. and Won et al. [36, 37]. Moreover, disabilities were more common in lower limbs. This led them to have difficulties doing common daily activities, maintaining hygiene, and walking. Rahim et al. [14] also stated that difficulties in doing these activities were due to the involvement of the lower limb (both soft-tissue and articular involvement).

Among these 143 patients of the present study, 41.9% of patients developed chronic CHIK arthritis. The 3-year longitudinal study conducted in La Reunion reported that 60% of patients experienced symptoms of arthralgia [39]. Long-term arthralgias were typically polyarthralgia (70%), usually symmetrical (90%). Chronic inflammatory arthritis was uncommon in the rural community of India (0·3% at one year) [15]. In Colombia, at least half of the patients developed chronic rheumatologic sequelae [40]. Chronic arthritic disability was documented 45%, where polyarthritis and arthralgia were common features in the village of Sri Lanka [36]. A systematic review and meta-analysis showed that the pooled prevalence of pCHIK-CIR at 18 selected studies among 5,702 patients was 40.2%. From studies derived from India, the prevalence was 27.3%, while the prevalence was 50.2% from France. The systematic review and meta-analysis of Rodriguez-Morales et al. reported that the prevalence of pCHIK chronic arthritis was 13.7% [12]. These discrepancies might result from different strains of CHIKV in other world regions. Alternatively, reported differences might result from the different genetic backgrounds of these populations. As joint pain is considered a subjective symptom, it might also reflect a difference in patients' pain threshold or reporting from physicians, thus reflecting differences in health care practices.

The majority had undifferentiated arthritis among these chronic patients, while 7 (11.7%) developed rheumatoid arthritis. Among the RA patients, two patients were Rheumatoid Factor positive (14.3%). Several authors reported variable reports regarding Rheumatoid factors in individuals who had CHIKV infection [15, 41, 42]. Manimunda et al. tested the Rheumatoid Factor in chronic patients where they found no patient with positive Rheumatoid Factor [43]. Anti-CCP was positive in two patients. However, the case report of Lynch and Pegler [44] reported that an individual with chronic CHIKV infection was anti-CCP negative. Though there is variability in Rheumatoid Factor and anti-CCP in different countries, this should not be ignored as these factors worsen the disabilities.

Tenosynovitis was present in 30.0% of patients of the present study. Other studies also found tenosynovitis as an early symptom [44–47]. Besides this, synovial hypertrophy and tendinitis were also found in some patients. Two patients developed bursitis, which is documented in literature as an early symptom [45, 46]. Three female and one male patient (12.5%) had median nerve entrapment, suggestive of carpal tunnel syndrome (CTS). Studies conducted in Sri Lanka and Thailand also found female preponderance regarding Carpal tunnel syndrome [36, 44].

The time of joint affection in CHIK infection is uncertain. Javelle reported that pCHIK-CIR could persist even after 6 years since acute infection in 59% of patients [11]. After one year, 21 patients of the present study had functional disabilities, where 11 had persistent pain for this long duration. In rural South India, 36.3% of patients were found to have persistent pain after 18 months [14]. One female and one male patient of the present study had a moderate functional disability which developed undifferentiated arthritis and spondyloarthritis. Severe functional disability was present in one female who had pre-existing spondyloarthritis. A history of the previous rheumatic musculoskeletal disorder increased the probability of severe disability by more than two times in patients with persistent pain. Post-CHIKV infection exerts more negative effects on the previous disease, contributing to severe disability [14]. As different studies found chronicity at different times, long-term follow-up should follow.

In the present study, patients with undifferentiated arthritis had mainly polyarthralgia. One-fourth had polyarthritis, while oligoarthralgia, monoarthritis, and oligoarthritis were also present in some patients. Rahim et al. found that patients with undifferentiated arthritis had monoarthritis [14]. Patients with spondyloarthritis, pre-existing arthritis, and rheumatoid arthritis had several joint involvements such as polyarthralgia or polyarthritis. After three months, less than half of the patients had polyarthralgia, and one-fifth had polyarthralgia & oligoarthritis. After six months, half of the patients had polyarthralgia, while at one year, polyarthralgia was present in 11 (52.4%) patients. Patients should be informed that return to regular activity would progressively occur in many cases. They could be benefitted from sharing information about the possible chronic symptoms and the unpredictable course of the disease.

Laise de Moraes et al. described a scoring system, SHERA (Sex, Hypertension, Edema, Retroocular pain, Age), to screen acutely CHIKV-infected patients at elevated risk of chronic arthralgia [48]. We found female gender, positive IgG, and moderate to severe functional disability as the contributory factor in chronicity development. None of our patients had retroocular pain.

The systematic review of van Aalst et al. female gender was a risk factor for chronification [49]. The present study also found a significant association between pCHIK-CIR and the female gender. Since most women spend most of their time in and around the home, interrupting vector habitats near houses might be a helpful way to control epidemics. A significant association was found between comorbidity and development of pCHIK-CIR, which was consistent with Schilte et al. [39]. Badawi et al. also reported that comorbidities like hypertension and diabetes might contribute to the severe outcome of CHIKV [50]. Patients who were IgG positive and had moderate to severe functional disability in the acute stage significantly developed pCHIK-CIR. Gerardin et al. identified severe rheumatic involvement (fever, at least six joints plus four other symptoms) at presentation and CHIKV-specific IgG titers as the main risk factors for relapsing or lingering rheumatic manifestations [51]. The association between chronicity and severity of the initial presentation was also reported in Mexican and Indian patients [52, 53]. All the patients with pre-existing RA had developed pCHIK-CIR. Moro et al. described the long-term clinical course and outcome of CHIKV following a Chikungunya (CHIKV) outbreak in Italy. They reported that persons with a history of rheumatologic diseases had higher anti-CHIKV IgG titers. At one year, the history of rheumatologic conditions was significantly associated with joint pain [54].

### Strength and limitations of the study

All the study subjects were followed up for one year. The rheumatologist confirmed the diagnosis. Only the confirmed cases of CHIKV infection were included. The sample size was relatively small. Therefore, musculoskeletal ultrasonography was not done in all patients.

## Conclusions

In conclusion, more than one-third of the CHIKV infected patients remained symptomatic after one year of the CHIKV outbreak in 2017. Polyarthralgia was the predominant clinical feature. Mild functional disability was also observed in a significant number of patients. Female patients with comorbidity, positive Ig G, moderate to severe functional disability, and pre-existing RA had significantly more chance of having pCHIK-CIR. Therefore, an integrated care plan should be made for patients with acute CHIK consisting of follow-up appointments with clinicians to identify pCHIK rheumatic disorders quickly and manage them accordingly. Above all, mass public awareness should be created to control the *Aedes* mosquito to reduce the disease burden.

## Data Availability

The datasets used and analyzed during the current study are available from the corresponding author on reasonable request.

## Abbreviations

ASAS: Assessment of Spondyloarthritis International Society
CHIKV: Chikungunya virus
CHIKF: Chikungunya fever
CI: Confidence interval
CTS: Carpal tunnel syndrome
DMARDs: Disease-modifying antirheumatic drugs
HAQ: Health assessment questionnaire
OR: Odds ratios
pCHIK-CIR: Post-chikungunya-chronic inflammatory rheumatism
RA: Rheumatoid arthritis
SpA: Spondyloarthritis
STROBE: Strengthening the Reporting of Observational Studies in Epidemiology
UA: Undifferentiated arthritis
WHO: World Health Organization
PDUS: Power doppler ultrasonography
